# The Prevalence of Colistin Resistant Strains and Antibiotic Resistance Gene Profiles in Funan River, China

**DOI:** 10.3389/fmicb.2018.03094

**Published:** 2018-12-18

**Authors:** Hongmei Tuo, Yanxian Yang, Xi Tao, Dan Liu, Yunxia Li, Xianjun Xie, Ping Li, Ju Gu, Linghan Kong, Rong Xiang, Changwei Lei, Hongning Wang, Anyun Zhang

**Affiliations:** Animal Disease Prevention and Food Safety Key Laboratory of Sichuan Province, Key Laboratory of Bio-Resource and Eco-Environment of Ministry of Education, College of Life Sciences, Sichuan University, Chengdu, China

**Keywords:** colistin, antibiotic resistance, *mcr-1*, *mcr-3*, urban river, quantitative polymerase chain reaction

## Abstract

Anthropogenic activities near urban rivers may have significantly increased the acquisition and dissemination of antibiotic resistance. In this study, we investigated the prevalence of colistin resistant strains in the Funan River in Chengdu, China. A total of 18 *mcr-1*-positive isolates (17 *Escherichia coli* and 1 *Enterobacter cloacae*) and 6 *mcr-3*-positive isolates (2 *Aeromonas veronii* and 4 *Aeromonas hydrophila)* were detected, while *mcr-2*, *mcr-4* and *mcr-5* genes were not detected in any isolates. To further explore the overall antibiotic resistance in the Funan River, water samples were assayed for the presence of 15 antibiotic resistance genes (ARGs) and class 1 integrons gene (*intI1*). Nine genes, *sul1*, *sul2*, *intI1*, *aac(6′)-Ib-cr*, *bla*_CTX-M_, *tetM*, *ermB*, *qnrS*, and *aph(3′)-IIIa* were found at high frequencies (70–100%) of the water samples. It is worth noting that *mcr-1*, *bla*_KPC_, *bla*_NDM_ and *vanA* genes were also found in water samples, the genes that have been rarely reported in natural river systems. The absolute abundance of selected antibiotic resistance genes *[sul1, aac(6′)-Ib-cr, ermB, bla_CTX-M_, mcr-1*, and *tetM]* ranged from 0 to 6.0 (log_10_ GC/mL) in water samples, as determined by quantitative polymerase chain reaction (qPCR). The *sul1*, *aac(6′)-Ib-cr*, and *ermB* genes exhibited the highest absolute abundances, with 5.8, 5.8, and 6.0 log_10_ GC/mL, respectively. The absolute abundances of six antibiotic resistance genes were highest near a residential sewage outlet. The findings indicated that the discharge of resident sewage might contribute to the dissemination of antibiotic resistant genes in this urban river. The observed high levels of these genes reflect the serious degree of antibiotic resistant pollution in the Funan River, which might present a threat to public health.

## Introduction

Multi-drug resistant (MDR) Gram-negative pathogens are resistant to almost all antibiotics, including cephalosporins, quinolones, aminoglycosides and carbapenems, making treatment difficult. Colistin is considered the last line of defense against MDR Gram-negative pathogens, playing an important role in the treatment of severe bacterial infections (Zavascki et al., 2007). Unfortunately, the recent emergence of plasmid-mediated colistin resistance genes in carbapenem-resistant *Enterobacteriaceae* presents a serious new threat to human health. The plasmid-mediated colistin resistance gene *mcr-1* was first discovered Liu et al. (2016). Soon afterward, another mobile phosphoethanolamine transferase gene, termed *mcr-2*, was discovered in porcine and bovine *Escherichia coli* isolates in Belgium (Xavier et al., 2016). Recently, Yin et al. (2017) discovered a novel *mcr* subtype, *mcr-3*, encoded on an IncI2 plasmid in an *E. coli* isolated from a pig in China. The *mcr-4* and *mcr-5* genes were detected in Europe almost simultaneously (Borowiak et al., 2017; Carattoli et al., 2017). Although there have been numerous reports of colistin resistance genes in animals and humans, fewer studies have focused on *mcr*-bearing isolates from aquatic environments.

Due to the continual release of antibiotic residues and antibiotic resistant bacteria (ARB) into the environment from hospitals, livestock facilities, and sewage treatment plants (STP), antibiotic resistant genes (ARGs) are regarded as environmental contaminants (Pruden et al., 2006; Zurfluh et al., 2017). The occurrence and dissemination of antibiotic resistance in pathogenic and zoonotic bacteria pose a potential threat to human health (Rosenberg Goldstein et al., 2012; Neyra et al., 2014). Moreover, an increasing number of bacteria are resistant to multiple antibiotics, and are able to transfer their resistant determinants among different bacterial species and genera in aquatic environments (Akinbowale et al., 2006). Urban rivers may provide an ideal setting for the acquisition and dissemination of antibiotic resistance because they are frequently impacted by anthropogenic activities. Although antibiotic resistance is a major and developing public health concern, the surveillance of this phenomenon in urban rivers is remarkably limited.

The Funan River, a major urban river in Chengdu used for agricultural activities (e.g., irrigation and cultivation) as well as recreational activities (e.g., swimming and fishing), was used as the model in this study to analyze the magnitude of antibiotic resistance in urban rivers.

The objectives of this study were: (1) to determine the prevalence of colistin resistance strains in the Funan River; (2) to investigate the MDR phenotypes and genotypes of isolated colistin resistant strains; (3) to screen for resistance determinants, including *sul1, sul2, bla*_CTX-M_, *bla*_V IM_, *bla*_KPC_, *bla*_NDM_, *qnrS*, *aac(6′)-Ib-cr*, *vanA*, *mecA*, *ermB*, *ermF*, *tetM, aph(3′)-IIIa*, and *mcr-1*, and the class 1 integron gene (*intI1*) in water samples from the Funan River.

## Materials and Methods

### Sampling of River Water

**FIGURE 1:**
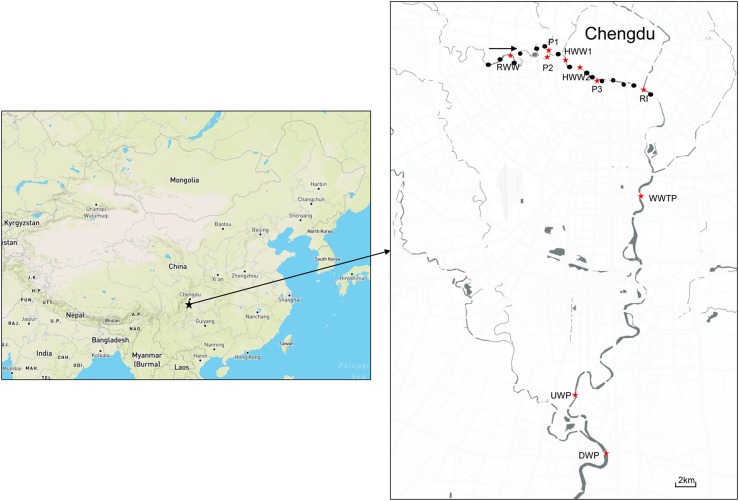
Study area with sampling sites to explore the antibiotic resistance of bacteria throughout the Funan River. Black dots indicate partial sampling sites for the detection of colistin resistant bacteria and red stars indicate sampling sites for the ARG determination in river water. (RWW, Residential Wastewater; WWTP, Municipal Wastewater Treatment Plant; P, Park; HWW, Hospital Wastewater; RI, River Intersection; UWP, Upstream of Wetland Park; DWP, Downstream of Wetland Park).

To investigate the prevalence of colistin resistant strains, 30 water samples (2 L) were collected from the Funan River near densely populated areas in September 2017. To further explore the antibiotic resistance of bacteria throughout the Funan River, 10 water samples (2 L) were collected from representative locations along the river (Figure 1). The representative locations included river intersections, streams near parks, and sewage outlets near residential areas, the hospital, and the municipal wastewater treatment plant (WWTP). The site near the residential sewage outlet is designated RWW and the sample near the municipal wastewater treatment plant is designated WWTP. Sites P1, P2, and P3 are close to various parks and HWW1 and HWW2 are close to the hospital sewage outlet. Site RI is located adjacent to the intersection of a tributary and the mainstream of the river. Sites UWP and DWP are upstream and downstream of Wetland Park, respectively. Water samples were collected from each site, immediately placed on ice, and transported to the laboratory within 4 h. The samples were then maintained at 4°C until investigation.

### Bacterial Isolation

A total of 30 water samples were concentrated by vacuum filtration through 0.22 μm filter membranes. The membranes were washed and the collected material was suspended in 10 ml of sterile PBS. A volume of 1 ml thereof was added to 9 ml of Brain Heart Infusion (BHI) broth with polymyxin B at a final concentration of 4 μg/mL. After incubation at 37°C overnight, 100 μl culture samples were streaked onto MacConkey agar plates. Fifty colonies were picked from each MacConkey agar plates and subsequently grown in BHI broth with 4 μg/mL polymyxin B for 18–24 h. Isolates were screened for the presence of *mcr-1, mcr-2, mcr-3, mcr-4*, and *mcr-5* by PCR. Next, *mcr*-positive isolates were purified by subculturing. The *mcr*-positive isolates were identified using 16S rRNA gene sequencing and the BD Phoenix-100 Automated Microbiology System (BD Diagnostic Systems, Sparks, NV, United States).

### Antimicrobial Resistance Testing and Detection of *mcr*-Positive Strains Genotype

The minimum inhibitory concentration (MIC) of colistin was determined by broth microdilution. The antimicrobial susceptibility was interpreted according to the guidelines of the European Committee on Antimicrobial Susceptibility Testing (EUCAST) version 6.0 (EUCAST, 2017). Fourteen antimicrobial agents were tested: ampicillin (AMP, 10 μg), amoxicillin/clavulanic acid (AMC, 20/10 μg), cefotaxime (CTX, 30 μg), ceftriaxone (CRO, 30 μg), ceftazidime (CAZ, 30 μg), cefoxitin (FOX, 30 μg), imipenem (IPM, 10 μg), ertapenem (ETP, 10 μg), aztreonam (ATM, 30 μg), ciprofloxacin (CIP, 5 μg), fosfomycin (FOS, 50 μg), tetracycline (TE, 30 μg), amikacin (AK, 30 μg) and trimethoprim/sulfamethoxazole (SXT, 1.25/23.75 μg). Antimicrobial susceptibility was determined by the agar disk diffusion method. Isolates were classified as susceptible, intermediate, or resistant using the breakpoints specified by the Clinical and Laboratory Standards Institute (CLSI) (CLSI, 2016). *Escherichia coli* ATCC 25922 was used as the quality control strain.

After DNA extraction using the TIANamp bacteria DNA kit (TIANGEN, China), the isolates were screened for the presence of 21 antibiotic resistance genes (*bla*_KPC_, *bla*_OXA-48_, *bla*_NDM_, *bla*_V IM_, *bla*_IMP_, *bla*_SHV_, *bla*_TEM_, *bla*_CTX-M-1_, *bla*_CTX-M-9_, *fosA3*, *qnrB*, *qnrS*, *floR*, *oqxAB*, *sul1*, *sul2*, *tetM*, *tetA*, *aac(6′)-Ib-cr*, *rmtA*, and *rmtB*) (Berendonk et al., 2015; Zheng et al., 2015; Liu et al., 2016), and the primers and PCR conditions used are listed in Table 1. Negative and positive controls for PCR of each gene were utilized.

**Table 1:** Standard primer pairs used in this study.

Target genes	Sequence (5′→3′)	Amplicon size(bp)	Reference
*mcr-1*	CGGTCAGTCCGTTTGTTC	350	Liu et al., 2016
	CTTGGTCGGTCTGTA GGG		
*mcr-2*	TGGTACAGCCCCTTTATT	1617	Xavier et al., 2016
	GCTTGAGATTGGGTTATGA		
*mcr-3*	TTGGCACTGTATTTTGCATTT	542	Yin et al., 2017
	TTAACGAAATTGGCTGGAACA		
*mcr-4*	ATTGGGATAGTCGCCTTTTT	487	Carattoli et al., 2017
	TTACAGCCAGAATCATTATCA		
*mcr-5*	ATGCGGTTGTCTGCATTTATC	1644	Borowiak et al., 2017
	TCATTGTGGTTGTCCTTTTCTG		
*bla*_ KPC_	ATGTCACTGTATCGCCGTC	902	Zheng et al., 2015
	TTACTGCCCGTTGACGCC		
*bla*_OXA-48_	TTGGTGGCATCGATTATCGG	744	Zheng et al., 2015
	GAGCACTTCTTTTGTGATGGC		
*bla*_ NDM_	ATGGAATTGCCCAATATTATGCAC	813	Zheng et al., 2015
	TCAGCGCAGCTTGTCGGC		
*bla*_V IM_	TTTGGTCGCATATCGCAACG	500	Zheng et al., 2015
	CCATTCAGCCAGATCGGCAT		
*bla*_ IMP_	GTTTATGTTCATACWTCG	432	Zheng et al., 2015
	GGTTTAAYAAAACAACCAC		
*bla*_SHV_	ATTTGTCGCTTCTTTACTCGC	861	Zheng et al., 2015
	TTTATGGCGTTACCTTTGACC		
*bla*_ TEM_	ATGAGTATTCAACATTTCCGTG	861	Zheng et al., 2015
	TTACCAATGCTTAATCAGTGAG		
*bla*_CTX-M_	TTTGCGATGTGCAGTACCAGTAA	759	Zheng et al., 2015
	CGATATCGTTGGTGGTGCCATA		
*bla*_ CTX-M-1_	AAAAATCACTGCGCCAGTTC	415	Zheng et al., 2015
	AGCTTATTCATCGCCACGTT		
*bla*_ CTX-M-9_	CAAAGAGAGTGCAACGGATG	205	Zheng et al., 2015
	ATTGGAAAGCGTTCATCACC		
*fosA3*	GCGTCAAGCCTGGCATTT	282	Hou et al., 2012
	GCCGTCAGGGTCGAGAAA		
*qnrB*	GATCGTGAAAGCCAGAAAGG	469	Wang et al., 2017
	ACGATGCCTGGTAGTTGTCC		
*qnrS*	ACGACATTCGTCAACTGCAA	540	Wang et al., 2017
	TAAATTGGCACCCTGTAGGC		
*oqxAB*	CCCTGGACCGCACATAAAG	1140	Wang et al., 2017
	AAAGAACAAGATTCACCGCAAC		
*sul1*	ATGGTGACGGTGTTCGGCATTCTG	840	Hur et al., 2011
	CTAGGCATGATCTAACCCTCGGTC		
*sul2*	GAATAAATCGCTCATCATTTTCGG	810	Hur et al., 2011
	CGAATTCTTGCGGTTTCTTTCAGC		
*tetM*	AGTGGAGCGATTACAGAA	158	Adefisoye and Okoh, 2016
	CATATGTCCTGGCGTGTCTA		
*tetA*	GCTACATCCTGCTTGCCTTC	210	Adefisoye and Okoh, 2016
	CATAGATCGCCGTGAAGAGG		
*aac(6′)-Ib-cr*	TTGCGATGCTCTATGAGTGGCTA	482	Eftekhar and Seyedpour, 2015
	CTCGAATGCCTGGCGTGTTT		
*rmtA*	CTAGCGTCCATCCTTTCCTC	635	Wang et al., 2017
	TTGCTTCCATGCCCTTGCC		
*rmtB*	GCTTTCTGCGGGCGATGTAA	173	Wang et al., 2017
	ATGCAATGCCGCGCTCGTAT		
*floR*	GTCGAGAAATCCCATGAGTTCA	1645	Cloeckaert et al., 2000
	CAGACAGGATACCGACATTCAC		
*intI1*	GGGTCAAGGATCTGGATTTCG	484	Mazel et al., 2000
	ACATGCGTGTAAATCATCGTCG		
*vanA*	AATACTGTTTGGGGGTTGCTC	734	Kafil and Asgharzadeh, 2014
	TTTTTCCGGCTCGACTTCCT		
*mecA*	TGGTATGTGGAAGTTAGATTGGGAT	155	Paterson et al., 2012
	CTAATCTCATATGTGTTCCTGTATTGGC		
*ermB*	GATACCGTTTACGAAATTGG	364	Zhang et al., 2016
	GAATCGAGACTTGAGTGTGC		
*ermF*	CGACACAGCTTTGGTTGAAC	309	Zhang et al., 2016
	GGACCTACCTCATAGACAAG		
*aph(3′)-IIIa*	GCC GAT GTG GAT TGC GAA AA	269	Udo and Dashti, 2000
	GCT TGA TCC CCA GTA AGT CA		

### Total DNA Extraction and Detection of ARGs

To further explore the extent of antibiotic resistance throughout the Funan River, water samples were collected from 10 locations (Figure 1). Total DNA was extracted using the Water DNA kit (OMEGA, United States) from the bacteria sample trapped by 0.22 μm pore filter (2 L samples). Standard PCR performed as listed in Table 1 was used to detect 15 ARGs (*sul1, sul2, bla*_CTX-M_, *bla*_V IM_, *bla*_KPC_, *bla*_NDM_, *qnrS*, *aac(6′)-Ib-cr*, *vanA*, *mecA*, *ermB*, *ermF*, *tetM*, *aph(3′)-IIIa* and *mcr-1*) and the class 1 integron gene (*intI1*). Negative and positive controls were used for each set of PCR primers. PCR amplification reactions were conducted in 20 μl volumes containing 1× PCR Master Mix (Tsingke, China), 1.0 μl template DNA, and 0.5 μM of each primer. After amplification, 5 μl samples of the PCR products were loaded on a 1.0% agarose gel containing GoldView, and separated electrophoretically in 1 × TAE buffer at 120 V for 20 min and visualized.

### Quantitative Polymerase Chain Reaction

To compare the abundance of ARGs for different sampling sites, the gene copy numbers of the *sul1*, *aac(6′)-Ib-cr*, *ermB*, *bla*_CTX-M_, and *tetM* genes were quantified using qPCR assays. These genes confer resistance to five major classes of antibiotics: sulphonamides, aminoglycosides, macrolides, β-lactams, and tetracyclines. The levels of *mcr-1* and 16S rRNA genes were also quantified. To quantitate the amounts of these genes, the levels were compared to the levels in standard samples prepared from plasmids containing these specific genes, as described previously (Chen and Zhang, 2013). The standard samples were diluted to yield a series of 10-fold concentrations and were subsequently used to generate qPCR standard curves. The *R*^2^ values were higher than 0.990 for all standard curves. The 20 μl qPCR mixtures contained 10 μL of SYBR premix Ex Taq^TM^ (TaKaRa, Dalian, China), 0.5 μM of each forward and reverse primer, and 1 μl of template DNA. The final volume was adjusted to 20 μl by addition of DNase-free water. The IQ^TM^5 real-time PCR system was employed for amplification and quantification, using the following protocol: 30 s at 95°C, 40 cycles of 5 s at 95°C, 30 s at the annealing temperature, and extension for another 30 s at 72°C. For detection, simultaneous fluorescence signal was scanned at 72°C, followed by a melt curve stage with temperature ramping from 65 to 95°C. Details of the qPCR primers of the target genes and the annealing temperatures are given in Table 2. The method design was adopted from prior research (Thornton and Basu, 2011). The copy numbers of the selected ARGs were normalized against the 16S rRNA gene copy number. Therefore, the copy number unit is described as copies/16S.

**Table 2:** Quantitative polymerase chain reaction primer pairs used in this study.

Target genes	Sequence (5′→3′)	Amplicon size(bp)	Annealing temperatures (°C)	Reference
*sul1*	CACCGGAAACATCGCTGCA	158	60	Luo et al., 2010
	AAGTTCCGCCGCAAGGCT			
*aac(6′)-Ib-cr*	GTTTCTTCTTCCCACCATCC	103	60	Yang et al., 2018
	AGTCCGTCACTCCATACATTG			
*ermB*	CACCGAACACTAGGGTTGC	129	55	This study
	TGTGGTATGGCGGGTAAGT			
*bla*_ CTX-M_	CAGATTCGGTTCGCTTTCAC	103	55	Yang et al., 2018
	GCAAATACTTTATCGTGCTGATG			
*mcr-1*	CATCGCGGACAATCTCGG	116	56	Yang et al., 2017
	AAATCAACACAGGCTTTAGCAC			
*tetM*	TTCAGGTTTACTCGGTTCA	106	55	This study
	GAAGTTAAATAGTGTTCTTGGAG			
*16S rRNA*	CGGTGAATACGTTCYCGG	128	55	Suzuki et al., 2000
	GGWTACCTTGTTACGACTT			

### Statistical Analysis

Statistical analysis was performed using SPSS 17.0 (IBM, United States). One-Way ANOVA was employed to analyze the results and values of *P* < 0.05 were considered statistically significant.

## Results and Discussion

### The Prevalence of *mcr*-Positive Isolates in the Funan River

The screening of 1500 isolates for *mcr* yielded a total of 24 *mcr*-positive isolates. They included 18 *mcr-1* positive isolates (17 *Escherichia coli* and 1 *Enterobacter cloacae*) and 6 *mcr-3* positive isolates (2 *Aeromonas veronii* and 4 *Aeromonas hydrophila)*. *mcr-2*, *mcr-4*, or *mcr-5* were not observed in any of the isolates.

Many reports have described the presence in *mcr-1* in animal- and human- derived *Enterobacteriaceae* isolates isolated worldwide (Du et al., 2016; Liu et al., 2016; Malhotra-Kumar et al., 2016; Shen et al., 2016), but only two previous studies identified *mcr-1* in waterborne *Enterobacteriaceae*. One study reported detection of the *mcr-1* gene in 1 out of 74 *Enterobacteriaceae* isolated from 21 rivers and lakes in Switzerland that produced extended spectrum β-lactamases (ESBLs) (Zurfuh et al., 2016). In a separate study, similar to our results, Zhou et al. (2017) isolated 23 *mcr*-1-positive isolates from environmental water sources in Hangzhou, indicating that *mcr-1*-carrying *Enterobacteriaceae* may be common in lakes and rivers in China. Data addressing the prevalence of *mcr-3* is limited. Recently, a novel *mcr* variant, *mcr-3*, was first discovered on an IncI2 plasmid from a strain of *E. coli* isolated from a pig in China (Yin et al., 2017). Since then, *mcr-3*-positive strains have been identified in humans and food (Ling et al., 2017; Liu L. et al., 2017). Worryingly, *mcr-3* has been detected on the chromosome of *Aeromonas veronii*, and these chromosomally encoded *mcr-3* determinants can become plasmid-bound and transferable (Cabello et al., 2017; Ling et al., 2017). Recently, Shen et al. (2018a) presented evidence that *mcr* determinants originated from aquatic environments, including *mcr*-3 harboring *Aeromonas* spp. Because *Aeromonas* species are prevalent in aquatic environments, the occurrence of colistin resistant isolates in urban rivers is of great concern as these strains may contribute to the potential dissemination of *mcr* determinants.

### Antimicrobial Resistance Phenotypes and Genotypes of *mcr-1* and *mcr-3*-Positive Strains

As shown in Table 3, we next analyzed the antimicrobial resistance phenotypes and genotypes of the isolated *mcr-1* and *mcr-3* positive strains, and found 21 (87.5%) multidrug resistance isolates. The antimicrobial resistance testing showed that all isolates were resistant to colistin (MIC ≥ 4 μg/mL). Of the other antimicrobials tested, the most frequent resistance was to CTX (75%, 18 isolates), followed by CAZ (50%, 12 isolates), AMP (50%, 12 isolates), CRO (45.8%, 11 isolates), ATM (45.8%, 11 isolates), SXT (41.7%, 10 isolates), FOS (29.2%, 7 isolates), TE (25%, 6 isolates), AK (20.8%, 5 isolates), CIP (20.8%, 5 isolates), IPM (16.7%, 4 isolates), FOX (12.5%, 3 isolates), AMC (12.5%, 3 isolates), and ETP (4.2%, 1 isolate). The high occurrence of ESBL producers is worrisome, and corresponds to Zurfluh et al. (2013) who found 74 ESBL-producing isolates from 21 (36.2%) of 58 rivers and lakes, and all showed the multidrug resistance phenotype. In another study, 70% of fluoroquinolone resistant *E. coli* isolated from an urban river showed resistance to three or more classes of antibiotics (Zurfluh et al., 2014). The widespread distribution of MDR bacteria suggested serious drug-resistant pollution in river water. In this study, cephalosporin resistant strains were found most frequently, which may be related to the extensive use of cephalosporins for clinical and veterinary purposes. Overall, high usage has led to increased occurrence and wide distribution of ESBLs in bacteria (Bradford, 2001; Bonnet, 2004).

**Table 3:** The antimicrobial resistance genotypes, phenotypes and MIC values of colistin of *mcr-1* and *mcr-3* positive strains.

Isolates	Species	Antibiotic resistant genes	Antimicrobial resistance phenotypes^a^	MIC values of colistin (μg/mL)
E22	*Escherichia coli*	*mcr-1*	CTX, CAZ, AMP, ATM	16
E23	*Escherichia coli*	*mcr-1*	CTX, CAZ	16
E24	*Escherichia coli*	*mcr-1*	CTX, CAZ	16
E25	*Escherichia coli*	*mcr-1*	CTX, CAZ, ATM	16
E26	*Escherichia coli*	*mcr-1*	CTX, CAZ, ATM, AK	16
E27	*Escherichia coli*	*mcr-1, sul2*	CRO, ATM, SXT	16
E28	*Escherichia coli*	*mcr-1*	CTX, CRO, CAZ, ATM, AK	16
E29	*Escherichia coli*	*mcr-1, bla*_CTX-M-9_*, fosA3, qnrS, floR, oqxAB, sul1, sul2, tetA, aac(6′)-Ib-cr*	CTX, CRO, AMP, SXT, CIP	16
E30	*Escherichia coli*	*mcr-1, sul1, tetA*	CRO, FOX, ATM, SXT, FOS, TE	16
E31	*Escherichia coli*	*mcr-1, floR, sul2, tetM*	CTX, CRO, CAZ, SXT	16
E32	*Escherichia coli*	*mcr-1, floR, sul2, tetM*	CTX, CRO, CAZ, SXT	16
E33	*Escherichia coli*	*mcr-1, floR, sul2, tetM*	CTX, CRO, CAZ, SXT	16
E34	*Escherichia coli*	*mcr-1, bla*_TEM_*, bla*_CTX-M-9_	CTX, CRO, CAZ, AMP	16
E35	*Escherichia coli*	*mcr-1, bla*_TEM_*, bla*_CTX-M-9_*, floR, oqxAB, sul1, sul2, tetM, tetA, aac(6′)-Ib-cr, rmtB*	CTX, CRO, FOX, AMP, ATM, SXT, TE, AK, CIP	16
E36	*Escherichia coli*	*mcr-1, bla*_TEM_*, bla*_CTX-M-9_*, fosA3, oqxAB, sul1, sul2*	CTX, CRO, AMP, ATM, SXT, FOS, CIP	16
E38	*Escherichia coli*	*mcr-1, tetM, tetA*	CTX, CAZ, AMP, ATM, TE	16
E39	*Escherichia coli*	*mcr-1, qnrS, tetA*	CTX, ATM, AMC, TE, FOS, CIP	8
E37	*Enterobacter cloacae*	*mcr-1, floR, sul2, rmtA, rmtB*	CTX, FOX, AMP, AMC, SXT, AK, IPM	16
A4	*Aeromonas veronii*	*mcr-3, bla*_SHV_*, sul1*	CTX, IPM	4
A19	*Aeromonas hydrophila*	*mcr-3, bla*_TEM_*, bla*_CTX-M-9_*, qnrB, sul1, sul2, tetA*	CTX, CRO, CAZ, AMP, ATM, AMC, FOS, TE, IPM	16
A48	*Aeromonas hydrophila*	*mcr-3, sul1, rmtA, rmtB*	AMP, FOS	8
A49	*Aeromonas hydrophila*	*mcr-3, sul1, sul2, rmtA, rmtB*	AMP, FOS, AK	8
A52	*Aeromonas hydrophila*	*mcr-3, qnrS, floR, sul1, tetA*	AMP, FOS, TE CIP	4
A54	*Aeromonas veronii*	*mcr-3, sul1*	AMP, SXT, IPM, ETP	4

^a^*CTX, cefotaxime; CRO, ceftriaxone; CAZ, ceftazidime; FOX, cefoxitin; AMP, ampicillin; ATM, aztreonam; AMC: amoxicillin-clavulanic acid; SXT, trimethoprim-sulfamethoxazole; FOS, fosfomycin; TE, tetracycline; AK, amikacin; CIP, ciprofloxacin; IPM, imipenem; ETP, ertapenem*.

The *mcr-1* and *mcr-3* positive isolates were next assayed for the presence of other ARGs. The *bla*_SHV_, *bla*_TEM_ and *bla*_CTX-M-9_ genes were detected in 1 (4.2%), 4 (16.7%), and 5 (20.8%) isolates, respectively. None of the isolates were positive for *bla*_KPC_, *bla*_OXA-48_, *bla*_NDM_, *bla*_V IM_, *bla*_IMP_ or *bla*_CTX-M-1_. Fifteen (62.5%) of isolates contain sulphonamide resistance genes (*sul1* in 5 isolates, *sul2* in 5 isolates, and *sul1*/*sul2* combined in 5 isolates). Some isolates contained genes encoding tetracycline resistance, with 20.8% and 29.2% positive for *tetM* and *tetA* genes, respectively. Some isolates contained genes encoding fluoroquinolone resistance genes, *qnrB*, *qnrS*, and *oqxAB*, which were detected in 1(4.2%), 3(12.5%), and 3(12.5%) isolates, respectively. Genes associated with aminoglycoside resistance, *aac(6′)-Ib-cr*, *rmtA*, and *rmtB*, were amplified in 2 (8.3%), 3 (12.5%), and 4 (16.7%) isolates, respectively. The *floR* gene was detected in 7 (29.2%) isolates and the *fosA3* gene was identified in 2 (8.3%) isolates. According to a recent report, 77.3% of *mcr-1*-positive *E. coli* (34/44) carried at least 1 ESBL gene, and several isolates carried 3 or more ESBL genes (Wu et al., 2018). Furthermore, *bla*_CTX-M-9_ was one of the most prevalent genes among the identified ESBL genes in China (Liu et al., 2015). Consistent with previous reports, sulphonamides and tetracycline resistance genes are the most abundant ARGs in rivers (Yang et al., 2018). We identified two strains (E29 and E36) that carried *mcr-1*, *fosA3*, and *bla*_CTX-M-9_ genes from river samples (Table 3). The *mcr-1*, *fosA3*, and ESBLs genes were previously identified in *E. coli* isolated from animal and food samples (Liu X. et al., 2017; Lupo et al., 2018), and the presence of these multidrug-resistant strains in urban river may present a serious threat to public health.

### Prevalence of Antibiotic Resistance Genes in the Funan River

In this study, the prevalence of ARGs in water samples was investigated by sampling various sites along the Funan River. The *sul1, qnrS, tetM*, and *intI1* genes were detected in samples from all 10 sampling sites (100%). Additionally, *aac(6′)-Ib-cr*, *sul2*, *aph(3′)-IIIa*, *ermB*, and *bla*_CTX-M_ were detected at high rates of 90%, 90%, 90%, 80% and 70%, respectively. Many studies have reported the presence of these genes in aquatic environments (Hu et al., 2008; D’Costa et al., 2011; van Hoek et al., 2011; Lin et al., 2015; Makowska et al., 2016). Interestingly, the *aph(3′)-IIIa* gene has rarely been reported in river water microorganisms, but has been reported in clinical specimens (Tuhina et al., 2016). The detection of the *aph(3′)-IIIa* gene was high in this study, suggesting contamination of the Funan River with resistant bacteria carrying the *aph(3′)-IIIa* gene.

Genes conferring resistance to the last line of antibiotics, including *mcr-1*, *bla*_NDM_, *bla*_KPC_ and *vanA* genes, were detected at rates of 30%, 20%, 10%, and 10%, respectively. *bla*_V IM_ was not detected at any site. The *mcr-1* gene was detected in 30% of samples, suggesting the Funan River could act as a reservoir for the *mcr-1* gene. The *bla*_NDM_, *bla*_KPC_ and *vanA* genes were detected near the WWTP (Figure 1). Although *mcr-1* is found frequently in human and animal settings, there is only limited data for urban rivers (Marathe et al., 2017; Ovejero et al., 2017; Yang et al., 2017). Similarly, Marathe et al. detected *bla*_NDM_ and *bla*_KPC_ genes in the sediments of an Indian river (Marathe et al., 2017). Although a *bla*_V IM_ positive carbapenem-resistant strain was isolated from a river in Switzerland (Zurfluh et al., 2013), here is a lack of data on *bla*_V IM_ in the non-clinical environment. The *vanA* gene is associated with vancomycin resistance and has been found in wastewater biofilms and in drinking water biofilms in Mainz (Schwartz et al., 2003). Although these genes have rarely been identified in natural aquatic environments, given the dangerous infections that can arise from ARB (and which subsequently create intractable challenges for clinical treatment), further observation of the prevalence of these genes in aquatic environments is required.

### Abundance of ARGs

**FIGURE 2:**
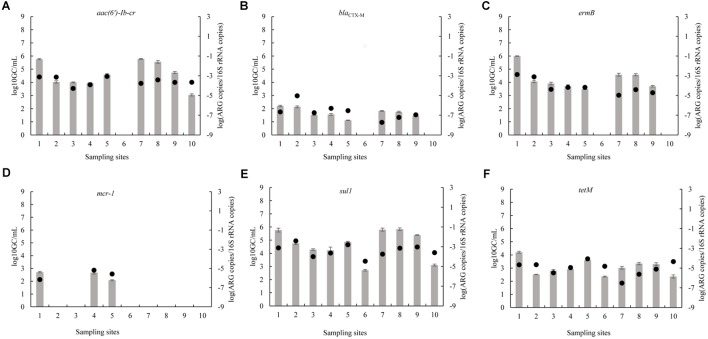
Absolute (bars) and 16S rRNA gene-normalized (symbols) levels of ARGs (**A**: *aac(6′)-Ib-cr*; **B**: *bla*_CTX-M_; **C**: *ermB*; **D**: *mcr-1*; **E**: *sul1*; **F**: *tetM*) in water samples collected at various sites (1, RWW, Residential wastewater; 2, P1, Park1; 3, P2, Park2; 4, HWW1, Hospital Wastewater1; 5, HWW2, Hospital Wastewater2; 6, P3, Park3; 7, RI, River Intersection; 8, WWTP, Municipal Wastewater Treatment Plant; 9, UWP, Upstream of Wetland Park; 10, DWP, Downstream of Wetland Park) along the Funan River.

Concerning the absolute abundance of ARGs in the Funan River, ARGs were detected at levels that ranged from 0 to 6.0 log_10_ GC/mL (Figure 2). The *sul1*, *aac(6′)-Ib-cr*, and *ermB* genes were the dominant ARGs in the Funan River with mean absolute abundances of 4.8, 4.1, and 3.4 log_10_ GC/mL, respectively. The *sul1* gene exhibited the most prominent average abundance in water samples. Previous studies reported that *sul1* is abundant in numerous water areas, including the Tordera River Basin (Proia et al., 2016) and the Haihe River (Luo et al., 2010). Although the *mcr-1* gene was not detected in water samples at some sites, three sites (RWW, HWW1, and HWW2) displayed 2.0-2.7 log_10_ GC/mL. Notably, the highest detected level of *mcr-1* (2.7 log_10_ GC/mL) was higher than that in previous reports about the Haihe river (2.6 log_10_ GC/mL) (Yang et al., 2017). The absence of *mcr* in some samples may indicate that no *mcr-1* positive strains were present in the water samples or that the levels of *mcr-1* were below the detection limit. Site RWW is located near the residential sewage outlet, suggesting the presence of *mcr-1* was related to human activity. Consistently, *mcr-1* was detected at HWW1 and HWW2, adjacent to the hospital sewage outlets, suggesting the spread of *mcr-1* from hospitals to urban river, although colistin is not used widely in human medicine. The *mcr-1* abundance at RWW (2.7 log_10_ GC/mL) was slightly higher than that at HWW1 (2.6 log_10_ GC/mL) and at HWW2 (2.3 log_10_ GC/mL). Similarly, the prevalence of *mcr-1*-positve *E. coli* from healthy individuals (0.7–6.2%) is higher than the prevalence for inpatients (0.4–2.9%) (Shen et al., 2018b). It is striking that *mcr* is the only gene that was absent from sites other than RWW and HWW. The reasons for high rate of fecal carriage of *mcr* in humans in China may reflect the rapid emergence of plasmid-encoded *mcr-1* within many MDR *E. coli* carried by humans and also be related to the significant diversity and genetic flexibility of MGEs harboring *mcr-1* (Zhong et al., 2018).

At RWW, RI, and WWTP, the absolute abundances of certain ARGs (*sul1*, *aac(6′)-Ib-cr*, and *ermB*) were significantly higher than those at other sampling sites (*P* < 0.05). At P3 and DWP, the absolute abundances of most ARGs were significantly lower than the levels detected at the other sites (*P* < 0.05). RWW was associated with the highest absolute abundance of the six ARGs (*mcr-1*, *sul1*, *aac(6′)-Ib-cr*, *ermB*, *bla*_CTX-M_, and *tetM*) (Figure 2). Samples near the wastewater treatment plant (WWTP) and densely populated areas exhibited a relatively greater content of resistant genes. Wastewater discharge may contribute to the spread of ARGs into the environment, thereby affecting the bacterial communities of the receiving river (Marti et al., 2013; Xu et al., 2015). Our results indicate that human activities influence the dissemination of resistance genes in the Funan River. Remarkably, the absolute abundances of most ARGs were low at the DWP sampling point, located downstream of the wetland park. This is consistent with a decrease in the ARGs levels of the effluents from a constructed wetland with a free surface flow (Liu et al., 2014).

As shown in Figure 2, the relative abundances of each ARG are only partly correlated with their absolute abundance. That is, although the absolute abundances of most ARGs at RWW, RI and WWTP were relatively high, their relative abundances were comparatively low. These differences may be related to the differences in the proportion of resistant bacteria to total bacteria at each site (Tao et al., 2014).

## Conclusion

This study describes 18 *mcr-1*-positive strains and 6 *mcr-3*-positive strains isolated from the Funan River, of which 87.5% were found to be MDR. The *sul1*, *sul2*, *intI1*, *aac(6′)-Ib-cr*, *bla*_CTX-M_, *tetM*, *ermB*, *qnrS* and *aph(3′)-IIIa* genes were abundant in the Funan River. Interestingly, the *mcr-1*, *bla*_KPC_, *bla*_NDM_, and *vanA* genes were detected, although these four resistance genes have rarely been found in natural river systems. Notably, the *mcr-1* gene was detected at a rate of 30%. Our results suggest urban activities may increase the prevalence of antibiotic resistance genes and demonstrate the current presence of drug-resistance pollution in the Funan River. The processes by which the dissemination of ARGs occurs in urban rivers should be the focus of future studies.

## Author Contributions

AZ designed the study. HT, DL, XX, and PL carried out the sampling work. HT, YY, XT, and JG performed the experiments. AZ, HT, RX, LK, and CL analyzed the data. AZ, HT, YL, and HW drafted the manuscript. All authors have read and approved the final manuscript.

## Conflict of Interest Statement

The authors declare that the research was conducted in the absence of any commercial or financial relationships that could be construed as a potential conflict of interest.
